# Java brucea and Chinese herbal medicine for the treatment of cholesterol granuloma in the suprasellar and sellar regions: A case report and literature review

**DOI:** 10.1097/MD.0000000000005930

**Published:** 2017-02-03

**Authors:** Zhe Sun, Yang Cao, Lin-zhu Zhai

**Affiliations:** a First clinical medical college of Guangzhou University of Chinese Medicine, Guangzhou, Guangdong,China; b Department of Oncology Center, the First Affiliated Hospital, Guangzhou University of Chinese Medicine, Guangzhou, Guangdong, China.

**Keywords:** cholesterol granuloma, Java brucea, suprasellar and sellar, traditional Chinese medicine

## Abstract

**Rationale:**

A cholesterol granuloma (CG) is usually found in the middle ear, papilla, orbits, petrous apex, and choroid plexus, but is highly uncommon in the skull. In spite of benign clinicopathological lesions, bone erosion can be seen occasionally in the patient with CG. The optimal treatment strategy is radical surgery, but complete excision is usually impossible due to anatomical restrictions and a risk of injury to the key structures located nearby. Here, we report a patient with CGs in the suprasellar and sellar regions who was successfully treated with Java brucea and Chinese herbal medicine.

**Patient concerns:**

A 31-year-old man presenting with progressive decreased vision in both eyes was analyzed.

**Diagnoses:**

A skull magnetic resonance imaging (MRI) scan showed a low-density tumor in the uprasellar and sellar regions and histopathological examination revealed a CG.

**Interventions:**

The patient was referred the surgery and radiotherapy. In the meantime, brucea soft capsules and herbal medicine combined were administered to him.

**Outcomes:**

The related clinical symptoms and signs resolved significantly after several months, as his therapy progressed. The patient showed no sign of recurrence during the treatment period. Furthermore, he was still alive and disease-free at 37 months of follow-up visit.

**Lessons:**

Overall, brucea soft capsules and a Chinese herbal formula treatment combined could be beneficial in improving the patient's quality of life with CG in the skull.

## 1 Introduction

A cholesterol granuloma (CG) is an infrequent intracranial neoplasm that is typically located in the middle ear, papilla, orbits, petrous apex, and choroid plexus.^[1–6]^ However, CGs in the suprasellar and sellar regions are uncommon and some lesions initially identified as craniopharyngioma by radiographic features are actually CGs based on intraoperative findings and postoperative histopathology.^[7,8]^ Their pathogenesis is unclear to date. The majority of researchers have proposed a theory of airway obstruction in the cells. A benign tumor is a cystic lesion that is formed as a result of a giant cell's response to cholesterol crystals and metabolized blood byproducts.^[8,9]^ In addition, there are several case reports of intracranial CGs related to familial hypercholesterolemia and high serum lipid levels.^[9]^ Although radical surgery is the primary treatment and results in long-term survival, an endoscopic approach or radiotherapy is also recommended. However, the efficacy of these treatments cannot be guaranteed due to the rarity of this disease and the high risks of recurrence, which are described in the current report. No therapeutic regimens have been established for CGs.

In recent years, Chinese herbal medicine (CHM) has increasingly proven to be effective and popular in the management of patients with neoplasms, particularly in patients who exhibit resistance, complications, and severe side effects of conventional treatment for several cancer types.^[10]^ The role of CHM on the following aspects of cancer therapy has been extensively explored: prevention of tumorigenesis, alleviation of adverse effects, enhancement of efficacy of conventional therapy, reduction of tumor recurrence and metastasis, and improvement of a patient's quality of life.^[11]^ However, the potential role of this formula in cancer therapy has not yet been clearly addressed by modern science. In addition, *Brucea javanica* in CHM is characterized as an antipyretic and detoxifying plant that is distributed throughout South East Asia and South China. Its fruits have potent antimalarial, anti-inflammatory, and antiviral effects with low toxicity and have also been widely used in the treatment of lung, prostate, and gastrointestinal cancers. Increasing attention has been paid to the remarkable antitumor activity of *B. javanica*. Current pharmacological research has identified the main anti-tumor ingredients in *B. javanica* to be tetracyclic triterpene quassinoids.^[12,13]^ Nonetheless, the detailed mechanisms of the effects of *B. javanica* on neoplasms, especially those of the brain, have never been explored.

Here, we report a case involving a 31-year-old, recurrence-free survivor of CGs in the suprasellar and sellar regions who was treated with brucea soft capsules and a Chinese herbal formula.

## 2 Case report

**Figure 1:**
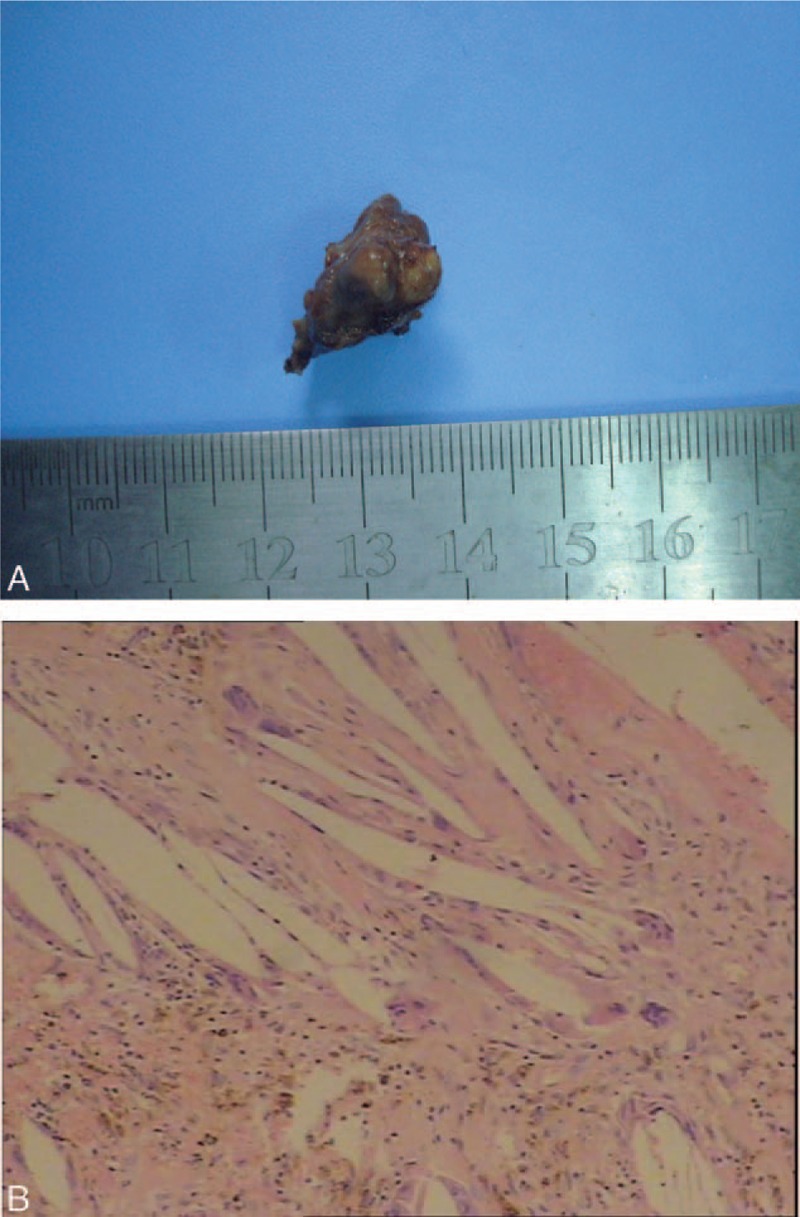
Macroscopic findings of resected tumor. Macroscopically, the tumor was ash—black, with the size of 2.5 cm × 1.5 cm × 1 cm (A). Microscopically, the tumor was characterized by numerous spindle-shaped cholesterol clefts, accompanied by foreign body giant cells, chronic inflammatory cells infiltrate, and hemorrhages with hemosiderin- laden (hematoxylin and eosin staining, original magnification, ×200) (B).

In March 2012, a 31-year-old man presented to a local hospital for progressive decreased vision in both eyes, along with dizziness, headache, nausea, and vomiting. An eye examination revealed that the vision in his left eye was 0.15 and that in his right eye was hand motion at 1 m. He did not have a genetic predisposition to familial hypercholesterolemia or hyperlipidemia and did not have a history of traumatic brain injury or a cerebral hemorrhage. Craniopharyngioma was suspected from the results of a skull magnetic resonance imaging (MRI) scan. In April 2012, he underwent a neoplasm resection. A histological examination of the surgical specimen showed a CG, which was contradictory to the MRI findings (Fig. 1). The final diagnosis was CG in the sellar and suprasellar regions. Unfortunately, in 2 months, he underwent conformal radiotherapy for a postoperative recurrence of the lesion. However, in October 2012, a brain MRI scan showed that the lesion was larger than that following radiotherapy. After receiving X-knife treatment, the patient was subsequently discharged.

On October 19th, the patient came to our outpatient clinic and presented with nausea and vomiting, poor binocular vision, radiating pain in the left lower extremity, and feeble limbs. Other findings of a physical examination included a reddish tongue with teeth prints and white greasy fur, and a wiry and thready pulse. In Chinese Medicine (CM) practice, he was diagnosed with a spleen deficiency and phlegm stagnation. Therefore, the therapeutic principle was to invigorate the spleen and remove phlegm. We prescribed a decoction by combining syndrome differentiation and disease differentiation, with 15 g of radix codonopsis preparata, 10 g of pericarpium citri reticulatae, 10 g of Rhizoma Pinellinae Praeparata, 30 g of poria cocos, 15 g of fructus aurantii immaturus, 10 g of caulis bambusae in taeniam, 15 g of vinegar rhizoma zedoariae, 15 g of rhizoma ligustici wallichii, 15 g of gastrodia elata, 15 g of grassleaf sweelflag rhizome, 15 g of Chinese taxillus twig, 20 g of Oldenlandia diffusa, 5 g of scorpio, 6 g of centipede, 20 g of Coicis semen, and 6 g of Radix Glycyrrhizae Preparata. The concoction was boiled in water and administered once a day. He followed this course of treatment for 28 days. After 3 courses of treatment, the therapeutic principle remained to invigorate the spleen and remove phlegm.

On January 11, 2013, the patient came in for a follow-up visit and told us that the pain in his left lower extremity had alleviated, but he still had nausea, headache, absence of poor binocular vision, and feeble limbs. His pulse was rough and he had a slightly purple tongue with stasis macules. In Traditional CM (TCM) practice, he was diagnosed with stasis of blood and phlegm. A decoction was prepared with 15 g of Angelica Dahurica, 3 g of taxus chinensis, 15 g of pericarpium citri reticulatae, 15 g of Rhizoma Pinellinae Praeparata, 30 g of poria cocos, 20 g of Coicis semen, 15 g of rhizoma ligustici wallichii, 15 g of salt cortex eucommiae, 5 g of safflower, 15 g of Parasitic loranthus, 30 g of Radix Fici Simplicissimae, 6 g of scorpion, 6 g of centipede, 15 g of vinegar rhizoma zedoariae, and 20 g of Oldenlandia diffusa. The decoction was administered once a day for about 6 months, according to a therapeutic principle for dissolving phlegm and activating blood stasis.

**Figure 2:**
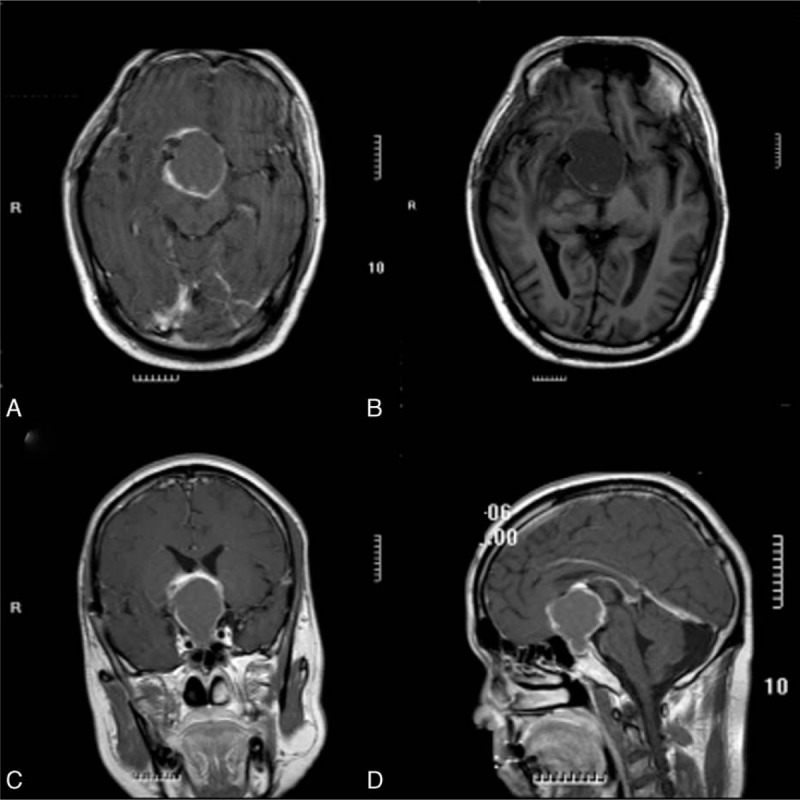
Magnetic resonance imaging showed cystosolid lesion with distinct border. The lesion was in size of 3.5 cm × 3.5 cm × 4.3 cm, with treatment of herbal decoction alone (August 2013) (A–D).

On August 2, 2013, the patient was admitted to our oncology department for memory deterioration, fatigue, and lethargy. His Karnofsky score was 40 and he was incapable of looking after himself. His MMSE (Mini Mental Status Examination) score, which detects changes in cognitive skills, was 14 (less than 17 points indicates severe cognitive impairment). His orientation, registration, attention, calculation, recall, language, and praxis were markedly reduced. An MRI scan of the skull revealed that the size of lesion was bigger than before (Fig. 2). As he declined to undergo surgery and radiotherapy due to financial constraints, he received an intravenous administration of a 30-mL brucea oil emulsion injection every day for 1 week. In addition, he was administered a TCM formula based on the aforementioned decoction (January 11, 2013) that also included 10 g of nidus vespae, 10 g of panax notoginseng, 15 g of gecko, and 30 g of Astragali Radix. The patient was discharged after his symptoms improved. He continued to go to an oncology outpatient clinic for treatment with a brucea soft capsule and a Chinese herbal decoction for 4 weeks. The Chinese herbal decoction contained 30 g of Astragali Radix, 20 g of Coicis semen, 15 g of pericarpium citri reticulatae, 15 g of Rhizoma Pinellinae Praeparata, 30 g of poria cocos, 15 g of rhizoma ligustici wallichii, 15 g of salt cortex eucommiae, 5 g of safflower, 15 g of Parasitic loranthus, 30 g of Radix Fici Simplicissimae, 6 g of scorpion, 6 g of centipede, 15 g of vinegar rhizoma zedoariae, 10 g of nidus vespae, and 15 g of gecko.

In September 2013, he was satisfied with the improvement in symptoms and signs; he also showed a Karnofsky score of 90 and an MMSE score of 28. In addition to the brucea soft capsules, a CM decoction based on the last formula was administered, excluding poria cocos, scorpion, and Chinese smartweed root and including 10 g of caulis bambusae in taeniam, 30 g of Astragali Radix, 15 g of fructus aurantii, 3 g of powdered notoginseng root, and 10 g of pseudobulbus cremastraeseu pleiones. The decoction was administered once daily for 7 courses (28 days per course) to remove phlegm and blood stasis and without replacement of the therapeutic principle. His symptoms disappeared gradually, with no recurrence during the treatment period.

**Figure 3:**
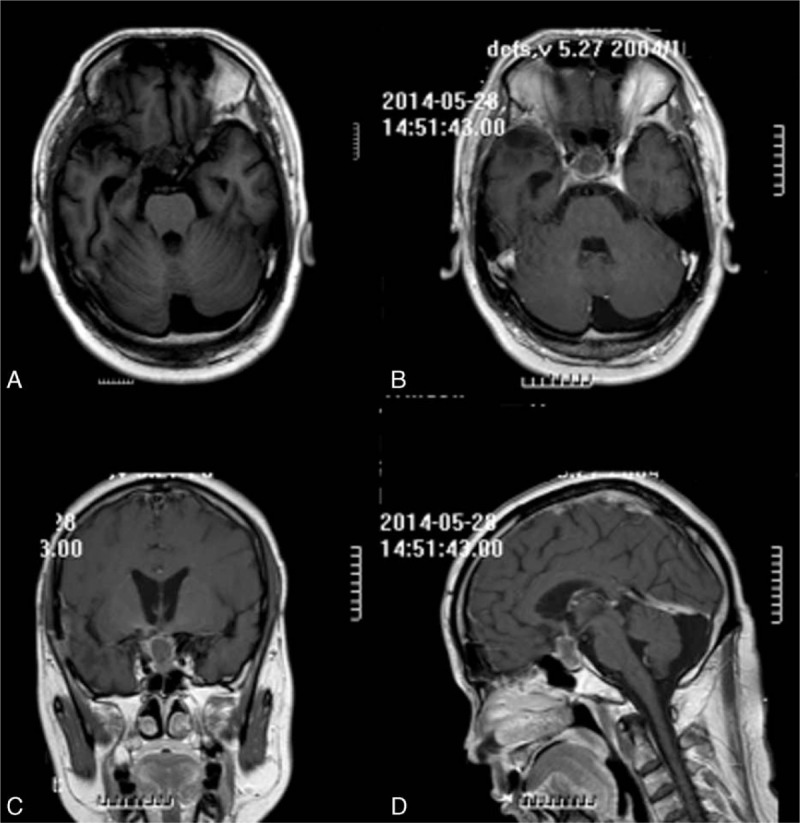
Magnetic resonance imaging findings revealed that the tumor size was 1.7 cm × 1.4 cm × 2.1 cm after Chinese herbal decoction combined with brucea soft capsules) (May 2014) (A–D).

**Figure 4:**
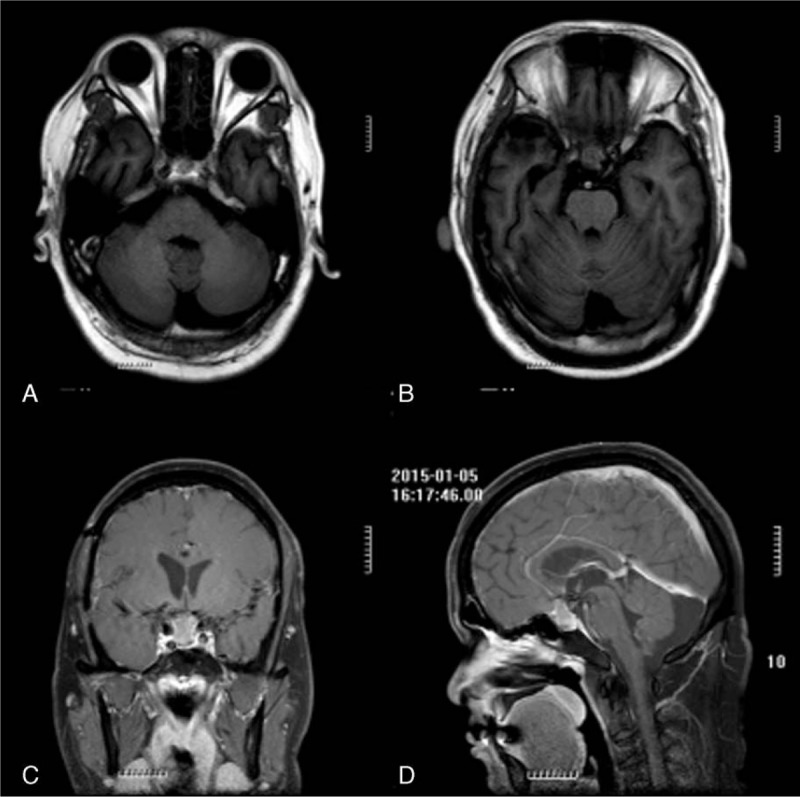
MRI suggested that the tumor size was 1.2 cm × 1.1 cm × 1.7 cm with the integrative therapy of traditional Chinese herbal decoction and brucea soft capsules (January 2015) (A–D).

**Figure 5:**
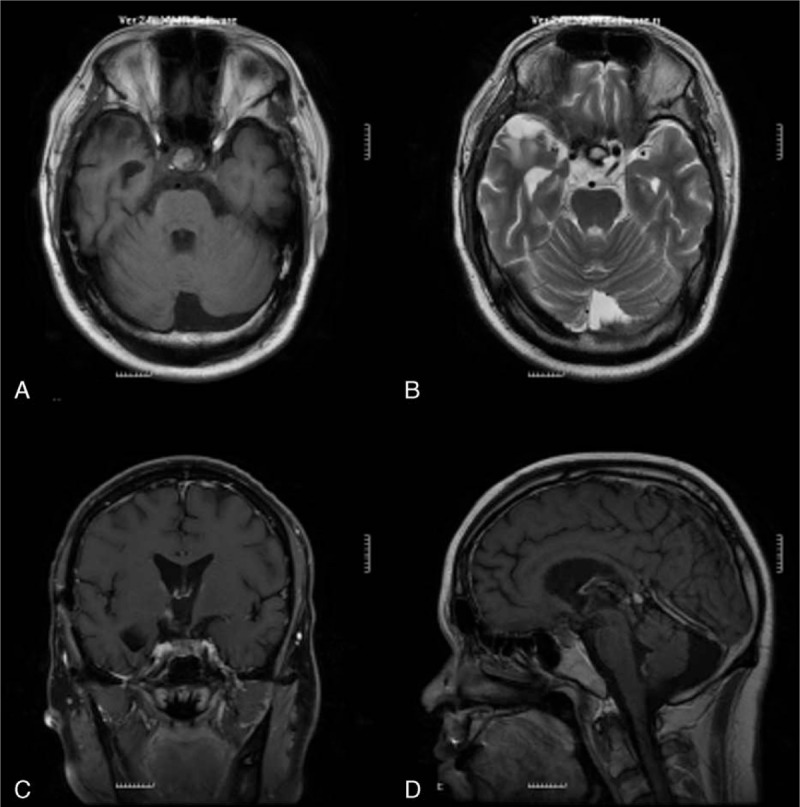
MRI showed the tumor size was 1 cm × 0.8 cm × 1.7 cm with the methods of brucea soft capsules and herbal medicine combined (September 2015) (A–D).

In May 2014, a brain MRI scan showed that the lesion was decreasing in size (Fig. 3). Thereafter, the patient was continuously treated with a brucea soft capsule and a herbal decoction for about 7 months. In January 2015, MRI scan results indicated that the lesion was smaller than that from the previous scan (Fig. 4). He visited us at regular intervals and underwent a brain MRI examination every 6 months. In September 2015, the most recent follow-up MRI scan showed further evidence of tumor reduction (Fig. 5). At present, the patient has continued to visit our oncology clinic and his health has improved gradually. He is asymptomatic with a Karnofsky score of 90, an MMSE score of 30, and normal levels of alanine amiotransaminase (ALT), aspartate aminotransaminase (AST), alkaline phosphatase (ALP), blood urea nitrogen (BUN), creatinine (Cr), and gamma glutamyl transferase (γ-GT). He showed no sign of recurrence during 37 months of follow-up.

## 3 Discussion

CGs located in the central nervous system are rare and are mostly associated with the petrous apex in the intracranial site.^[9]^ However, CGs in the suprasellar and sellar regions are uncommon; hence, there is very little literature on them. Computed tomography and MRI results suggest that they become enlarged by repeated hemorrhages and reparative tissue responses within the pituitary adenoma or are distinct forms of craniopharyngioma.^[9]^ The most suggestive symptoms of CGs in the suprasellar and sellar regions are not observable symptoms such as severe progressive headache, vomiting, vision loss, and drowsiness. Therefore, a final diagnosis relies on intraoperative findings and postoperative histopathology. CGs most commonly occur in men, with a male-to-female incidence ratio of 3:1, and among middle-aged patients.^[14,15]^

CGs are cystic lesions formed from metabolism of blood byproducts and foreign-body giant cell responses to a large quantity of cholesterol crystals.^[16–19]^ At present, they are thought to occur as a result of long-term chronic inflammation, infection, and surgical trauma history. However, CGs in the skull that do not originate from the petrous apex are pathologically distinct from CGs deriving from the petrous apex, which have no relevant association with inflammation and infection.^[20]^ This suggests that the risk factors differ among intracranial CGs from different sources. Previous case reports indicate that patients diagnosed with familial hypercholesterolemia or hyperlipidemia had asymptomatic manifestations, while intracranial CGs were not found to be associated with familial hypercholesterolemia or hyperlipidemia.^[9]^ In this case report, the patient did not have a history of a traumatic brain injury or cerebral hemorrhage. Furthermore, the pathogenesis of CG is unclear and speculative to date; however, most hypotheses suggest that an airway obstruction followed by drainage obstruction leads to the rupture of blood vessels and a hemorrhage. In this circumstance, red blood cell metabolic product accumulation may contribute to the formation of cholesterol crystals provoking a foreign-body giant cell reaction.^[16–19,21]^ A CG in the skull may occur as a consequence of a hemorrhage into the diploe of the bone.^[22]^

A CG is essentially a benign lesion, and because of a lack of typical distinguishing radiographic features, the treatment strategy is controversial. For patients who are asymptomatic, who have no abnormal findings following a physical examination of the nervous system, or who are in a stable condition, a long-term follow-up using imaging tests is recommended.^[16–20]^ In spite of a benign clinicopathological entity, bone erosion can be seen occasionally and enlarged lesions compressing adjacent tissue structures may cause a series of symptoms and signs, including a headache, nausea, vomiting, declining eyesight, and loss in memory. An optimal treatment strategy for these symptomatic cases is a total tumor resection, which would help lead to a definitive diagnosis based on the postoperative pathological results.^[23,24]^

In general, the chances of a tumor recurrence in the cases with complete excision are rare, but complete excision is usually impossible due to anatomical restrictions and a risk of injury to the key structures located nearby.^[12,14]^ Studies by Brackmann and Toh^[16]^ also reported that the incidence of postoperative recurrence was 14.7% in 34 patients with CG.^[12]^ In the other 17 case reports, a study by Hoa et al^[25]^ found that 41% of patients that underwent surgery had to undergo a second surgery.

Our case report showed that the patient relapsed after undergoing surgery, conformal radiotherapy, and gamma knife radiosurgery, which may have been induced by the nidus of deficiency of dermal layers and complex anatomical structures of tumor location.^[21]^ Currently, although open surgery is still common, most strategies use endoscopic techniques to remove the tumor, which is likely to reduce the risk but may also decrease the recurrence rate.^[26]^

The patient declined the above conventional therapies and opted for a traditional CM treatment. Due to financial constraints, he could not afford any western medicine therapies. During the period of outpatient treatment, the clinical symptoms and signs disappeared progressively. In addition, the lesion size also gradually narrowed. These results demonstrated the usefulness of decoction and Java brucea fruit soft capsule synergistic effects, further indicating that TCM played a vital role in the recovery of the patient.

In summary, a systematic review of the holistic treatment period suggests that a patient may obtain very good curative effects with TCM alone. First, we should emphasize a combination of tonification and purgation as well as a treatment regime for improving the body's resistance to eliminate pathogenic factors. The advantages of a TCM therapy for cancer are a holistic concept and treatment based on syndrome differentiation. Thereby, according to the therapeutic principles, we regard the local tumor lesions and the whole body as significant in the treatment of cancer. Second, the therapeutic principles of syndrome differentiation and disease differentiation should also be combined. We can use several antitumor herbal medicines, according to the location and characteristics of the tumor cells. Naturally occurring substances found in CHM are promising candidates that exert effective tumor chemoprevention and chemotherapeutic properties.

Despite the limited data, we can speculate that some components (Radix Astragali, Coicis semen, Oldenlandia diffusa, and Zedoary) in CHM may contribute to its efficacy in treatment of a CG. A large number of modern studies have revealed the anti-cancer mechanisms of CHM.^[10,11,27,28]^ For example, Radix Astragali, the main herb in our formula, is widely used as an immunomodulating agent in the treatment of many types of cancer.^[29–31]^ Total Astragalus saponins extracted from Radix Astragali may inhibit cell growth of various cancer cell lines via regulation of cell proliferation and apoptosis.^[32,33]^ In the colon, Astragalus saponins is likely to attenuate tumor cell growth through downregulation of vascular endothelial growth factor (VEGF) production, which modulates mammalian target of rapamycin and Cyclooxygenase 2 signaling.^[34]^ Coicis semen possesses antitumor and immune-enhancing properties, such as promoting T lymphocyte proliferation. Moreover, modern medicine research has established that coix seed emulsion from Coisis semen combined with gemcitabine had a much greater antitumor effect than the effect of either agent alone in pancreatic cancer through abrogation of nuclear factor-kappa B signaling.^[35]^ Another 2 components (Zedoary and Oldenlandia diffusa) also exhibit potential anticancer effects and restore the sensitivity of MCF-7/ADR (doxorubicin-resistant MCF-7 breast cancer cells) and A549/Taxol cells, modulating the multidrug resistance phenotype and the function of P-glycoprotein in human breast cancer cells.^[36,37]^

In this case report, other ingredients of CHM, including a scorpion, centipede, and gecko, have also been identified as effective anticancer drugs as prescriptions or formulas. They are classified as medical insects of well-known TCM. For example, a scorpion has been used in TCM to treat chronic neurological disorders, such as neuropathic pain, paralysis and epilepsy, for thousands of years. In the latest research, polypeptide extract from scorpion venom (PESV) may inhibit the angiogenesis of Lewis lung carcinomas (LCCs) by decreasing the level of VIII, alpha-SMA, DII4, and Notch1 in the tumor microenvironment.^[38]^ Furthermore, centipede and gecko, 2 types of traditional CM with remarkable antineoplastic activity, have been reported to induce cell cycle arrest and the apoptosis of cancer cells.^[39,40]^

A review of the complete therapy course of this case demonstrates that Brucea soft capsules play a key role in the process of recovery. It has been reported that the advantages of *B. javanica* include antitumor activity, low toxicity, and enhancement of immunity.^[12,13]^

*B. javanica* oil (BJO), extracted from dry fruit of CHM, is a complex mixture of fatty acids and their derivatives. Its main components are oleic acid and linoleic acid.^[9]^ Modern pharmacological research has demonstrated that tetracyclic triterpene quassinoids are the main antitumor ingredients in *B. javanica*, which may induce cancer cell death by various mechanisms, including the induction of apoptosis, regulation of the cell cycle, reversal of multidrug resistance, and a reduction in cell proliferation by reducing the expression of the *Bcl-2* gene.^[12,13,41–45]^ A report showed that *B. javanica* may inhibit Ehrlich ascites cancer cells in mice and ascites hepatoma cells in vitro.^[46]^

Although *B. javanica* is becoming increasingly popular in the treatment of lung, prostate, and gastrointestinal cancers and glioma, as well as in the reduction of intracranial pressure, it is not known whether these mechanisms involve a CG in the brain.

We speculate that *B. javanica* may exert efficiency, as pharmacokinetic studies have shown that approximately 90% of lipophilic emulsion particles with a diameter of 1 μm can cross the blood–brain barrier rapidly, enter the brain or cerebral tissue, and reach the location of lesion or metastasis,^[47,48]^ finally producing an effective concentration in the brain.^[49,50]^ Nonetheless, we have to admit that there is no direct evidence to explain the efficacy of *B. javanica* and the CM in the treatment of a CG.

However, TCM is mainly applied as complementary Chinese traditional patent medicine in the treatment of cancer and it has not been generalized to be applied to the treatment of malignant tumors. Furthermore, we believe that traditional CHM may contribute to the improvement of patient's quality of life and may be a good antineoplastic agent for benign tumors, especially cerebral tumors.

## 4 Conclusion

This case report shows that CGs in the suprasellar and sellar regions are rare, and a novel combination of a Java brucea fruit soft capsule and Chinese herbal decoction may have an antineoplastic effect with an excellent safety profile, which merits more attention.
